# *Ganoderma lucidum* culture supplement ameliorates dyslipidemia and reduces visceral fat accumulation in type 2 diabetic rats

**DOI:** 10.1080/21501203.2020.1740409

**Published:** 2020-03-23

**Authors:** Chung-Hsiung Huang, Wei-Kang Lin, Shun‐Hsien Chang, Guo-Jane Tsai

**Affiliations:** a Department of Food Science, National Taiwan Ocean University, Keelung, Taiwan; b Institute of Food Safety and Risk Management, National Taiwan Ocean University, Keelung, Taiwan; c Center for Marine Bioscience and Biotechnology, National Taiwan Ocean University, Keelung, Taiwan

**Keywords:** *Ganoderma lucidum*, hypolipidemic effect, lipid metabolism, submerged culture, type 2 diabetes, visceral fat accumulation

## Abstract

Diabetic rats were daily fed with a high-cholesterol diet containing 1% or 3% freeze-dried whole submerged *G. lucidum* culture or its mycelia for 5 weeks. Body weight, adipose tissue weight and plasma triglyceride levels were reduced, while high-density lipoprotein-cholesterol levels were elevated in rats fed with *G. lucidum* powder supplement diets. Notably, *G. lucidum* supplements downregulated the activities of hepatic acetyl-CoA carboxylase, fatty acid synthase and lipoprotein lipase, but upregulated the activity of hormone-sensitive lipase in the perirenal adipose tissues. Moreover, *G. lucidum* supplements increased the faecal triglyceride excretion. Therefore, daily supplementation of submerged *G. lucidum* culture, especially mycelia, can ameliorate dyslipidemia and reduce visceral fat accumulation in diabetic rats fed with a high-fat diet, which is closely related to the modulation of lipid synthesis, metabolism, and excretion.

## Introduction

Type 2 diabetes is a chronic metabolic disease with hyperglycaemia and insulin resistance. It is mainly caused by obesity and accounts for 90% of diabetes cases. (Wu et al. 2014). The symptoms of type 2 diabetes usually develop slowly. As a result, complications such as cardiovascular disease have often been associated with the diagnosis of diabetes (Grundy et al. 1999). Increasing evidence suggests that lipid metabolism and glucose metabolism are equally important in the development of diabetes. Visceral fat is thought to be the culprit in the development of insulin resistance, which plays an important role in the pathogenesis of dyslipidemia in type 2 diabetes (Patel and Abate 2013). Dyslipidemia is a well-recognised and modifiable risk factor and should be detected early to prevent cardiovascular diseases (Nelson 2013). Type 2 diabetes is associated with abnormal plasma lipids and lipoproteins, including decreased high-density lipoprotein cholesterol (HDL-C), predominant low-density lipoprotein cholesterol (LDL-C), and elevated triglyceride (TG) levels. These dyslipidemia characteristics increase the risk of cardiovascular disease (Nelson 2013). Increased liver secretion of very low-density lipoprotein (VLDL) and impaired clearance, leading to its conversion to LDL, are crucial in the pathophysiology of dyslipidemia (Krauss 2004). Although proper diet, exercise, and weight management may improve diabetic dyslipidemia and body fat accumulation, pharmacological treatment is still needed (Krauss 2004).

*Ganoderma lucidum* is a well-known medicinal mushroom that has been used as a health supplement (Galor et al. 2011). *G. lucidum* is widely consumed because it has various medicinal properties including antitumor, immune regulation, liver protection, antioxidant, anti-ageing, hypoglycaemic and hypolipidemic effects (Galor et al. 2011). In recent decades, *G. lucidum* has attracted great interest from scientists due to its potential as an alternative adjuvant therapy for diabetes. Both carbohydrate and lipid metabolism are targets of *G. lucidum* for improving the outcome of diabetes. For example, 26-oxysterols isolated from *G. lucidum* fruiting bodies showed an inhibitory effect on cholesterol synthesis (Hajjaj et al. 2005). Lanostane triterpenes harvested from *G. lucidum* fruiting bodies suppressed 3T3-L1 adipocytes differentiation (Lee et al. 2010). Genetic resources of Mexican *G. lucidum* showed cholesterol-lowering properties in mice fed a high-cholesterol diet (Meneses et al. 2016). The extracts of *G. lucidum* fruiting bodies, spores, and a proteoglycan PTP1B inhibitor isolated from *G. lucidum* also showed hypolipidemic activities in diabetic rodents (Wang et al. 2012, 2015; Bach et al. 2018). Moreover, water extracts of mycelia of *G. lucidum* have been reported to reduce the weight of the body, liver, and adipose tissue of mice fed a high-fat diet (Chang et al. 2015). At the same time, the results of clinical studies showed that *G. lucidum* could improve the occurrence of diabetic dyslipidemia (Chu et al. 2012). Therefore, it has been considered as a potential therapeutic candidate for lipogenic diseases.

Ganoderma fungus is composed of fruiting body (the basidiocarp), mycelium and spores (Galor et al. 2011). Compared with the cultivation of fruiting bodies and spores, the submerged *G. lucidum* culture has the advantages of lower production and time cost, less space requirements, easy-to-control culture conditions, and higher yield, purity and regeneration ability. (Galor et al. 2011). However, the components and their relative proportions in *G. lucidum* from basidiocarp cultivation are different from those in submerged culture (Galor et al. 2011).

As the effects of submerged *G. lucidum* culture on blood lipid and visceral fat accumulation are unknown, we used a type 2 diabetic rat model to deepen the understanding of the ameliorating effects of submerged *G. lucidum* cultures and their mycelia on hyperlipidaemia and visceral fat accumulation. Streptozotocin (STZ)-induced diabetic rats were daily fed with a high-cholesterol diet containing freeze-dried whole submerged *G. lucidum* cultures or mycelia for 5 weeks. Body weight and adipose tissue weight as well as TG, total cholesterol (TC), HDL-C, LDL-C, and VLDL-C levels were measured. Since dyslipidemia and visceral fat accumulation are involved in lipid synthesis, metabolism and excretion, the activities of key enzymes and faecal excretion of TG and TC were studied to clarify the underlying mechanism behind the beneficial effects of submerged *G. lucidum* cultures.

## Materials and methods

### Culture, chemicals and reagents

*G. lucidum* BCRC 36123 was obtained from Bioresources Collection and Research Centre (Hsinchu, Taiwan). All chemicals and reagents were purchased from Sigma-Aldrich Chemical Co. (St. Louis, MO, USA) unless otherwise stated. AIN-93 vitamin mixture and AIN-93 mineral mixture were purchased from MP Biomedicals LLC (Santa Ana, CA, USA). The rat insulin enzyme-linked immunosorbent assay kit was purchased from Randox Laboratories, Ltd. (Crumlin, UK), and the glucose detection kit was purchased from Audit Diagnostics (Cork, Ireland).

### Submerged culture of G. lucidum and sample preparation

Pieces of mycelium pad (0.5 x 0.5 cm^2^) of *G. lucidum* BCRC 36123 grown on the Gano medium (2.4 g glucose, 0.6 g yeast extract in 100 mL deionised water) agar plate at 30°C for 7 days were put into 1 mL frozen preserving medium (10 mL glycerol, 2 g dextran, 2 g trehalose dehydrate in 90 mL deionised water) for storage at −80°C. The frozen pieces of *G. lucidum* mycelium pad inoculated on Gano medium agar plates were incubated at 30°C for 7 days and two mycelial pieces (1 x 1 cm^2^) from the culture plate were added into a flask containing 250 mL Gano medium and incubated at 30°C, 110 rpm for 7 days. Based on the method described by Chang et al. (2006), the extracellular polysaccharides and mycelia in cultures were separated and measured. Whole submerged cultures and collected mycelia were freeze-dried and stored at −20°C for further experiments.

### Animals and experimental design

Male Sprague Dawley rats (8 weeks old) were purchased from BioLASCO Taiwan Co., Ltd (Taipei, Taiwan). The rats were housed in stainless steel cages with diet and water provided *ad libitum* in a temperature- and humidity-controlled animal room. All animal experiments were approved by the Institutional Animal Care and Use Committee of the National Taiwan Ocean University, and the rats were handled and euthanised in accordance with its guidelines.

**Figure 1.**
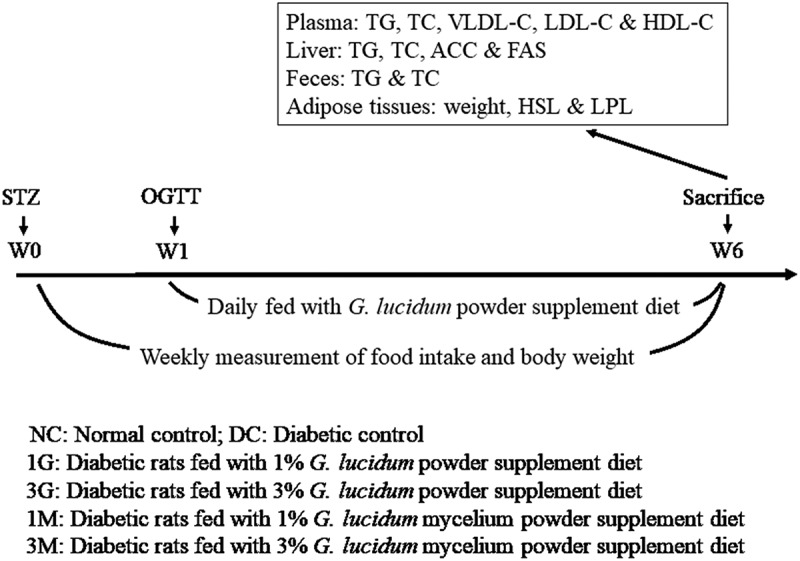
Protocols of type 2 diabetes induction and *G. lucidum* supplement diets treatment

On arrival, rats were fed with normal diet for 1 week and then randomly divided into six groups (n = 8): normal control (NC), diabetic control (DC), and four experimental groups including diabetic rats fed with 1% or 3% freeze-dried whole submerged culture of *G. lucidum* powder supplement diet (1G or 3G), and diabetic rats fed with 1% or 3% freeze-dried submerged culture of *G. lucidum* mycelia powder supplement diets (1M or 3M). The rats for diabetic control and experimental groups were subcutaneously injected with nicotinamide (230 mg/kg B.W.) and STZ (65 mg/kg B.W.) for the induction of type 2 diabetes (Masiello et al. 1998). One week later, oral glucose tolerance test was performed to confirm the successful induction of diabetes (Figure S1), and the rats were then daily fed with a high-cholesterol diet containing 1G, 3G, 1M, or 3M for 5 weeks. The composition of employed diets is shown in Table 1. Body weight and adipose tissue weights were measured, and samples of blood, faeces, liver, and perirenal and epididymal adipose tissues were harvested after sacrifice for further analysis (Figure 1).

**Table 1.** Composition of experimental diets

Ingredient (%)	NC/DC^1^	1/3G^1^	1/3M^1^
Casein	20	20	20
Lard	7	7	7
Soybean	1	1	1
Vitamin mixture^2^	1	1	1
Salt mixture^3^	4	4	4
Cholestrol	0.5	0.5	0.5
Choline chloride	0.2	0.2	0.2
Cholic acid	0.2	0.2	0.2
Cellulose	5	5	5
Corn Starch	61.1	61.1	61.1
*Ganoderma lucidum* powders	-	1/3	-
*Ganoderma lucidum* mycelium powders	-	-	1/3
Total	100	101/103	101/103

^1^NC: Normal control.

DC: Diabetic control.

1G: Diabetic + 1% freeze-dried *Ganoderma lucidum* culture powders.

3G: Diabetic + 3% freeze-dried *Ganoderma lucidum* culture powders.

1M: Diabetic + 1% freeze-dried *Ganoderma lucidum* mycelium powders.

3M: Diabetic + 3% freeze-dried *Ganoderma lucidum* mycelium powders.

^2^AIN-93 vitamin mixture.

^3^AIN-93 mineral mixture.

Sprague-Dawley (SD) rats were injected with nicotinamide and streptozotocin (STZ) to induce type 2 diabetes. After 1 week, oral glucose tolerance test (OGTT) was performed to verify the successful induction of diabetes. The rats in the normal control (NC) group did not receive any treatment throughout the experiment. Diabetic rats were randomly divided to 5 groups (n = 8), including diabetic control fed with high-cholesterol diet (DC), diabetic rats fed with high-cholesterol diet containing 1% freeze-dried whole submerged culture of *G. lucidum* (1G), diabetic rats fed with high-cholesterol diet containing 3% freeze dried whole submerged culture of *G. lucidum* (3G), diabetic rats fed with high-cholesterol diet containing 1% freeze dried mycelia from submerged culture of *G. lucidum* (1M) and diabetic rats fed with high-cholesterol diet containing 3% freeze dried mycelia from submerged culture of *G. lucidum* (3M). Except for the rats in NC and DC groups, the other rats were daily fed with *G. lucidum* supplement diets daily for 5 weeks as described in **Materials and methods**. The amount of food intake and body weight were measured weekly. All rats were sacrificed at week 6 to harvest the samples of blood, faeces, liver and adipose tissues for further analysis

### Determination of triglyceride (TG), total cholesterol (TC), and lipoprotein-cholesterol levels in the plasma

Blood samples were collected at sacrifice for the isolation of plasma by centrifugation (1,570 × g at 4°C for 20 min). Plasma concentrations of TG and TC were measured using enzymatic kits (Randox Laboratories Limited, UK) according to the manufacturer’s instructions.

A modified phosphotungstic acid/MgCI_2_ precipitation procedure was employed to isolate VLDL-C, LDL-C, and HDL-C (Assmann et al. 1983). The concentrations of cholesterol in the HDL-C-containing supernatants and VLDL-C- and LDL-C-containing precipitations were measured using enzymatic kits (Randox Laboratories Limited, UK) according to the manufacturer’s instructions to evaluate the plasma levels of HDL-C and LDL-C.

### Determination of TG and TC levels in the liver and faeces

Rat liver tissues were mixed with chloroform/methanol solution (2:1; *v/v*) and homogenised (Hong Sheng, SA-50 Max. 3000, Taipei, Taiwan) for 1 min. Supernatants were harvested by centrifugation (1,570 × *g* for 10 min) for lipid acquisition (Folch et al. 1957) and mixed with Triton X-100 followed by solvent removal using vacuum evaporation (SC 110; Savant Instruments, Holbrook, NY, USA.). TG and TC levels were measured using enzymatic kits (Randox Laboratories Limited, UK) according to the manufacturer’s instructions.

Faecal samples were dried following the method described in the Association of Official Analytical Chemists and ground into powder. The procedures of lipid extraction from dried faecal powder and determination of TG and TC levels were performed using the same method as that for the liver tissues.

### Evaluation of the activities of acetyl-CoA carboxylase (ACC) and fatty acid synthase (FAS) in the liver

Activities of ACC and FAS were measured and calculated as described previously (Ahmad et al. 1981). Briefly, liver samples were homogenised in citrate buffer/tri-sodium citrate solution. After centrifugation (10,600 × g at 4°C for 30 min), ACC activity was measured by mixing the supernatant with Tri-HCl buffer, MgCl_2_, monohydrate potassium citrate, glutathione, KHCO_3_, bovine serum albumin, acetyl-CoA, and ATP, followed by NADPH, and incubation for 5–6 s. Absorbance was measured at 340 nm under 37°C in 30-s intervals for 5 min using a UV–vis spectrophotometer (UV-7800, JASCO Co., Tokyo, Japan) to calculate the activity of ACC. FAS activity was measured by mixing the supernatant with K_2_HPO_4_, dithiothreitol, acetyl-CoA, EDTA‧2Na, and malonyl-CoA, followed by NADPH, and incubation for 5–6 s. Absorbance was measured at 340 nm under 37°C in 30-s intervals for 5 min using a UV–vis spectrophotometer. The optical density values were then used to calculate the specific activities of ACC and FAS according to the formula [V1 ÷ (ε × d × FP × V2)] × (ΔA/Δt) × 1000, where V1 represents the total reaction volume (2.9 mL), ε represents the molecular absorption coefficient (6.22 mL/μmol‧cm), d represents the optical path (1 cm), FP represents the protein concentration of the enzyme (mg protein/mL), V2 represents the volume of the enzyme (0.1 mL), and ΔA/Δt represents the change in absorbance of the enzyme reaction solution per unit time.

### Evaluation of hormone-sensitive lipase (HSL) and lipoprotein lipase (LPL) activity in perirenal adipose tissues

HSL activity was evaluated using methods based on the study of Morimoto et al. with minor modifications (Morimoto et al. 2001). Briefly, isolated perirenal adipose tissues were washed with 0.9% saline, cut up, and mixed with TES buffer followed by incubation at 37°C and 50 rpm in a shaking incubator. After incubation for 1, 2, and 3 h, samples were transferred into Eppendorf Microcentrifuge tubes and further incubated in a 70°C water bath for 10 min followed by centrifugation (100 × *g* at 25°C for 30 s). Lipolysis rate of the supernatant was measured using glycerol assay kits (Randox Laboratories Limited, UK) according to the manufacturer’s instructions.

LPL activity was evaluated using methods based on previous studies with minor modifications (Murase et al. 1981; Quinn et al. 1982; Shirai and Jackson 1982). Briefly, isolated perirenal adipose tissues were washed with 0.9% saline, cut up, and then mixed with Krebs-Ringer bicarbonate buffer, followed by incubation at 37°C and 50 rpm in a shaking incubator. After incubation for 1 h, the incubated samples were mixed with enzyme reaction solution and substrate solution. After incubation at 37°C for 10 min, the incubated solution was mixed with methanol/chloroform/heptane (10:9:7, v/v/v) and incubated at 42°C for 3 min followed by centrifugation (1,500 × *g* for 5 min). Absorbance of the supernatant was measured at 400 nm, and the optical density value was used for the calculation of LPL activity according to the Beer–Lambert law.

### Statistical analysis

All data were expressed as the mean ± standard error of the mean for each treatment group. Independent sample *t*-test was used to assess the statistical difference between the treatment groups and the DC control. All *p*-values <0.05 were considered statistically significant.

## Results

### Daily supplementation with a submerged culture of G. lucidum ameliorated hyperlipidaemia and visceral fat accumulation in diabetic rats fed with a high-cholesterol diet

**Figure 2.**
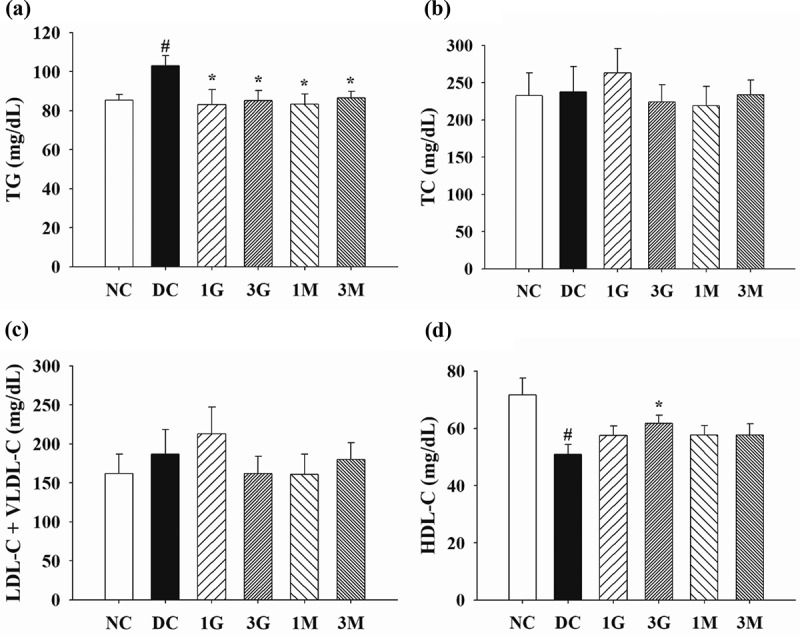
The concentrations of (a) triglyceride (TG), (b) total cholesterol (TC), (c) low density lipoprotein-cholesterol (LDL-C) and very low density lipoprotein-cholesterol (VLDL-C) and (d) high density lipoprotein-cholesterol (HDL-C) in the plasma of diabetic rats fed with *G. lucidum* supplement high-cholesterol diets

Plasma concentrations of TC and TG were measured to verify the hypolipidemic activities of *G. lucidum* supplement diets. Plasma TG level was significantly (*p* < 0.05) elevated in diabetic rats fed with a high-cholesterol diet compared with that in the NC group (Figure 2(a)). However, supplementation of the high-cholesterol diet with submerged culture of *G. lucidum* or mycelia significantly (*p* < 0.05) decreased the plasma TG concentration (Figure 2(a)). Although the plasma concentrations of TC and LDL-C+ VLDL-C were comparable between each group (Figure 2 (b,c)), supplementation with 3% whole submerged *G. lucidum* culture significantly (*p* < 0.05) reversed the high-cholesterol diet-induced HDL-C reduction in diabetic rats (Figure 2(d)). Based on the above data, daily supplementation with submerged *G. lucidum* cultures exhibited preventive effects against high-cholesterol diet-induced plasma TG elevation and HDL-C reduction in rats with type 2 diabetes.

The rats were treated as described in **Materials and methods**. After treatment for 5 weeks, the blood samples were collected to detect the concentrations of TG, TC, LDL-C+ VLDL-C, and HDL-C in plasma. Results are expressed as mean ± SEM for each group of rats (n = 8). ^#^*p* < 0.05 compared with the normal control (NC); *p < 0.05 compared with diabetic control (DC).

**Figure 3.**
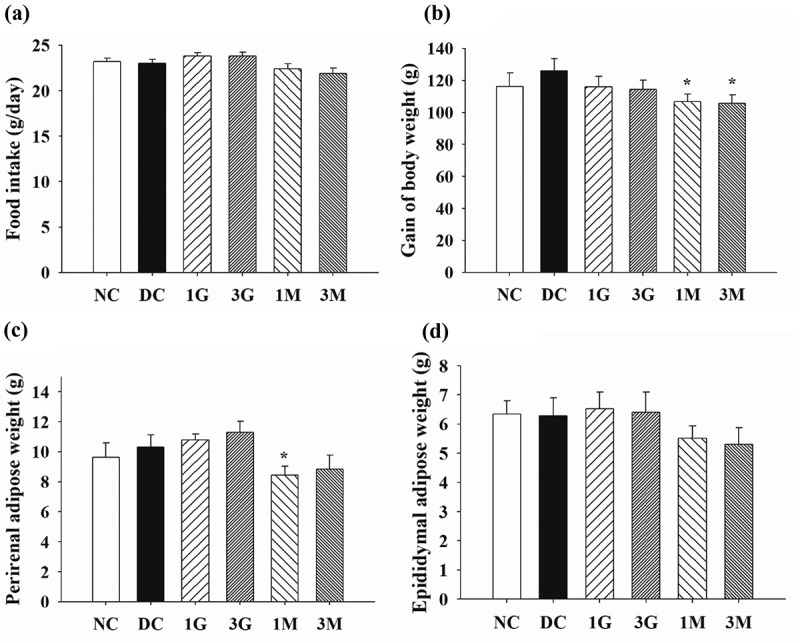
The amount of (a) food intake, (b) gain of body weight and weights of (c) perirenal and (d) epididymal adipose tissues of diabetic rats fed with *G. lucidum* supplement high-cholesterol diets

In order to explore the effect of *G. lucidum* supplement diets on the attenuation of body fat accumulation, the amount of food intake as well as body weight (B.W.) gain and adipose tissue weight were measured. Throughout the animal experiment, the amount of food intake was comparable between each group (Figure 3(a)). Nonetheless, B.W. gain was significantly (*p* < 0.05) reduced in the 1M and 3M groups compared with those in the DC group (Figure 3(b)). Consistent with these findings, decreasing weights of perirenal and epididymal adipose tissues were observed in rats in the 1M and 3M groups (Figure 3 (c,d)). These results indicate that daily dietary supplementation with mycelia harvested from submerged *G. lucidum* cultures attenuated B.W. gain and visceral fat accumulation in diabetic rats fed with a high-cholesterol diet.

The rats were treated as described in **Materials and methods**. The amount of food intake and body weight increment were measured weekly throughout the entire animal experiment. After sacrifice, the perirenal and epididymal adipose tissues were isolated and weighted individually. Results are expressed as mean ± SEM for each group of rats (n = 8). ^#^*p* < 0.05 compared with the normal control (NC); *p < 0.05 compared with diabetic control (DC).

### Daily dietary supplementation with the submerged culture of G. lucidum modulated lipid synthesis/metabolism enzyme activity and lipid excretion in diabetic rats fed with a high-cholesterol diet

**Figure 4.**
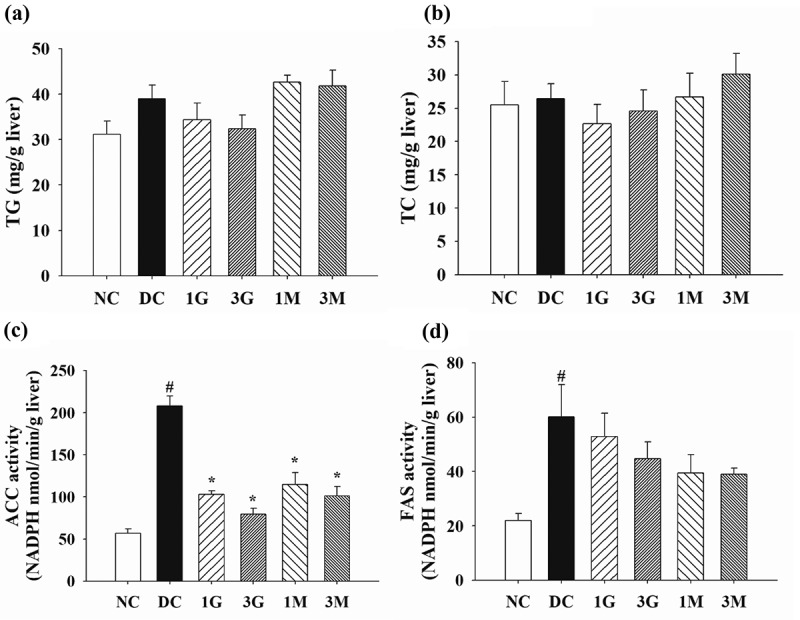
The concentrations of (a) triglyceride (TG) and (b) total cholesterol (TC) and the activities of (C) acetyl-CoA carboxylase (ACC) and (d) fatty acid synthase (FAS) in the liver of rats fed with *G. lucidum* supplement high-cholesterol diets

In order to elucidate the mechanism behind *G. lucidum*-induced hypolipidemic effects and prevention of visceral fat accumulation, we further measured the activities of lipid synthesis/metabolism enzymes and the level of lipid excretion. First, TG and TC concentrations and the activities of lipid synthesis enzymes in the liver were investigated. Although the concentrations of TG and TC were comparable between each group (Figure 4 (a,b)), the activities of ACC and FAS were significantly elevated in diabetic control rats (Figure 4 (c,d)). Notably, dietary supplementation with *G. lucidum* downregulated the activities of ACC and FAS (Figure 4 (c,d)), especially the ACC activity being marked reduced compared to that of DC group, indicating the suppressive effect of *G. lucidum* on lipid synthesis.

After treatment of *G. lucidum* supplement diets for 5 weeks, the rats were sacrificed to isolate the liver samples for the detection of TG, TC, FAS and ACC as described in **Materials and methods**. Results are expressed as mean ± SEM for each group of rats (n = 8). ^#^*p* < 0.05 compared with the normal control (NC); *p < 0.05 compared with diabetic control (DC).

**Figure 5.**
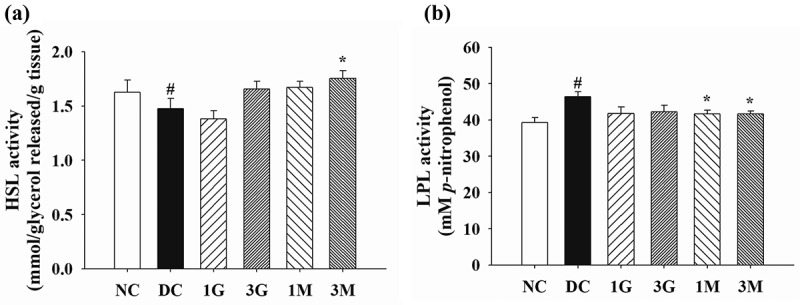
The activity of (a) hormone sensitive lipase (HSL) and (b) lipoprotein lipase (LPL) in the perirenal adipose tissues of diabetic rats fed with *G. lucidum* supplement high-cholesterol diets

On the other hand, the impact of *G. lucidum* on lipid metabolism in perirenal adipose tissues was elaborated by investigating the activities of HSL and LPL. A markedly reduced HSL activity and a significantly elevated LPL activity were observed in the perirenal adipose tissues of rats in the DC group compared with those in the NC group (Figure 5). Noticeably, HSL activity was significantly upregulated in the 3M group, whereas LPL activity was significantly downregulated in the 1M and 3M groups compared with those in the DC group (Figure 5 (a,b)). These findings suggest that dietary supplementation with mycelia harvested from submerged *G. lucidum* cultures potentially induced intracellular TG hydrolysis and prevented TG storage in adipose tissues by modulating HSL and LPL activities, respectively.

After treatment of *G. lucidum* supplement diets for 5 weeks, the rats were sacrificed and the perirenal adipose tissues were isolated individually for the measurement of HSL and LPL activities as described in **Materials and methods**. Results are expressed as mean ± SEM for each group of rats (n = 8). ^#^*p* < 0.05 compared with the normal control (NC); *p < 0.05 compared with diabetic control (DC).

**Figure 6.**
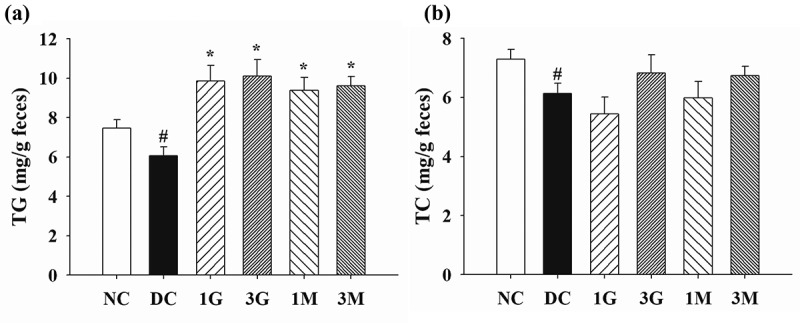
The levels of (a) triglyceride (TG) and (b) total cholesterol (TC) in the faeces of rats fed with *G. lucidum* supplement high-cholesterol diets

Finally, the faecal concentrations of TG and TC were investigated to understand the effect of *G. lucidum* on lipid excretion. We observed that the faecal concentrations of TG and TC were markedly reduced in diabetic rats compared with those of rats in the NC group (Figure 6). Dietary supplementation with *G. lucidum* significantly increased the faecal concentration of TG compared with that in the DC group (Figure 6(a)). Consistently, rats in the 3G and 3M groups had higher faecal concentrations of TC than those in the DC group, although no statistical difference was observed between the treatment groups and the DC group (Figure 6(b)). Our findings suggest that daily dietary supplementation with *G. lucidum* could reverse diabetes-induced reduction of lipid excretion, particularly that of TG, via the faecal route.

After treatment of *G. lucidum* supplement diets for 5 weeks, the faecal samples were collected individually for the detection of TG and TC concentrations as described in **Materials and methods**. Results are expressed as mean ± SEM for each group of rats (n = 8). ^#^*p* < 0.05 compared with the normal control (NC); *p < 0.05 compared with diabetic control (DC).

## Discussion

Although beneficial effects of *G. lucidum* fruiting bodies on obesity and dyslipidemia have been reported, limited information is available pertaining to the impact of submerged *G. lucidum* cultures on abnormal lipid metabolism. In the present study, we demonstrated the ameliorating effect of submerged *G. lucidum* cultures on dyslipidemia and visceral fat accumulation in diabetic rats fed with a high-cholesterol diet. This notion has been substantiated by several lines of evidence. Firstly, dietary supplementation with whole submerged culture of *G. lucidum* or its mycelia for 5 weeks downregulated the plasma TG levels and upregulated the plasma HDL-C levels. Secondly, B.W. and adipose tissue weight gains were decreased in rats fed with a mycelia supplement diet. Moreover, the potential mechanism of action behind the effects of submerged *G. lucidum* cultures was elucidated and supported by reduced activities of ACC and FAS in liver, reduced activities of LPL and enhanced activities of HSL in adipose tissue, as well as increased faecal excretion of TG. Our data clearly substantiate the beneficial effects of submerged *G. lucidum* cultures and their mycelia on high-cholesterol-induced abnormal lipid metabolism in rats with type 2 diabetes and provide critical insights into its underlying mechanisms of action.

Several lipid metabolising enzymes with different expression and activity have been identified between healthy individuals and patients with diabetes. These enzymes are also considered to be key factors that cause dyslipidemia and visceral fat accumulation. For example, ACC catalyzes the first step in the carboxylation of acetyl CoA to form malonyl CoA, which is then catalysed by FAS to synthesise fatty acids. Increasing evidence suggests that reducing ACC activity is necessary to reduce lipid accumulation and improve insulin action (Mao et al. 2006; Choi et al. 2007; Abu-Elheiga et al. 2012; Cordonier et al. 2016). (2007). Consistent with our results, Abu-Elheiga et al. (2012) and Choi et al. (2007) observed reductions in fat, lean body mass, and serum non-esterified fatty acids level, and prevention from diet-induced obesity in the ACC2 gene deleted (ACC2^−/-^) mice. In addition, ACC2^−/-^ mice were protected from fat-induced systemic and hepatic insulin resistance. It has also been reported that treatment of murine 3T3-L1 preadipocytes with soraphen A, ACC1 and ACC2 inhibitors prevents the accumulation of fat in adipocytes (Cordonier et al. 2016). Therefore, inhibition of ACC activity is a viable therapeutic target for the treatment of obesity (Moraski et al. 2013). Moreover, FAS also plays an important role in lipogenic pathway, which is related to the causal relationship between the consequences of excessive energy intake and the development of obesity and type 2 diabetes (Berndt et al. 2007). Our data show that in diabetic rats supplemented with *Ganoderma* diet, ACC activity was significantly reduced, while FAS activity was slightly reduced, suggesting that inhibition of lipid synthesis, especially ACC activity, is one of the potential mechanisms of beneficial effects of G. *lucidum* on dyslipidemia and visceral fat accumulation.

Related to lipid metabolism, HSL plays a key role in the hydrolysis of TG in various tissues, including adipose tissue. It has been reported that HSL activity is reduced in adipose tissue of patients with type 2 diabetes, and HSL protein expression is reduced in obese insulin-resistant states (Watt et al. 2005; Jocken et al. 2007). On the other hand, LPL, a well-known enzyme in TG-rich granules and HDL metabolism, is a determinant of plasma TG and HDL concentrations (Taskinen 1987). As our results indicate the modulation of HDL-C levels and HSL and LPL activity, the acceleration of lipid metabolism is considered to be another potential mechanism behind the beneficial effects of supplementing the *Ganoderma* diet. Furthermore, increased faecal TG levels were observed in diabetic rats fed with a *G. lucidum* supplement diet. Accordingly, we suggest that increased TG excretion into the faeces is another mechanism of action behind the beneficial effects of *G. lucidum*. Taken together, the ameliorating effects of submerged *G. lucidum* cultures and their mycelia on dyslipidemia and visceral fat accumulation are closely associated with their modulatory effects on lipid synthesis, metabolism, and excretion.

Except for 26-oxysterol, lanostane triterpenes and proteoglycan PTP1B inhibitors, there is limited information on the role of *G. lucidum* in regulating lipid metabolism (Hajjaj et al. 2005; Lee et al. 2010; Wang et al. 2012). In this study, compared to submerged *Ganoderma* culture, *Ganoderma* mycelium had higher efficacy in improvement of body weight and adipose tissue weight and HSL and LPL activity, while the whole submerged *Ganoderma* culture had a better effect on regulating plasma HDL-C levels. Both *Ganoderma* culture and its mycelia had similar efficacy in regulating plasma TG levels, ACC activity, and faecal TG excretion. These findings indicate that not only mycelium but also other active ingredients, such as extracellular polysaccharides, can also help improve the ameliorating effects of submerged *G. lucidum* cultures on abnormal lipid metabolism. Although many studies have shown that medical mushrooms have multiple pharmacological activities, these literature use crude extracts, including undetermined metabolite mixtures, whose efficacy *in vitro* is far from being standardised due to widely differing extraction and treatment methods (Chen et al. 2018; Girometta 2019). For submerged *Ganoderma* sp., *in vivo* experiments and pharmacological studies of the identified compounds are also very limited. Therefore, there is an urgent need to develop *in vivo* and pharmacological analysis methods for known compounds used in medical mushrooms. In order to clarify possible adverse reactions and mechanisms of action, it is necessary to have a better understanding of the bioactive compounds in *Ganoderma* sp., which will be beneficial for clinical applications (Basnet et al. 2017). Recently, Ogbole et al. (2019) have identified and authenticated a puffball mushroom using molecular tools and investigated its anti-diabetic properties of crude extract and partitioned fractions *in vitro* and *in vivo*. By using a similar strategy, further research is needed to identify the active ingredients that contribute to the regulation of lipid metabolism by submerged *G. lucidum* cultures.

## Conclusion

Our findings provide the first evidence that the improvement of dyslipidemia and visceral fat accumulation in type 2 diabetic rats fed with high-cholesterol diets by submerged *G. lucidum* culture and its mycelium in the diet may be through regulating lipid synthetic/metabolic enzyme activities and lipid excretion. Since submerged cultures have advantages over basidiocarp cultivation and have promising activity against abnormal lipid metabolism, we recommend using submerged *G. lucidum* culture and its mycelium as functional foods for controlling type 2 diabetes, especially for diabetes with complications of lipid metabolism disorder.

## Supplementary Material

Supplemental Material

## Data Availability

The data used to support the findings of this study are available from the corresponding author upon request.
