# Supplementary material for: Ganoderma lucidum culture supplement ameliorates dyslipidemia and reduces visceral fat accumulation in type 2 diabetic rats
Source: Mycology. 2020 Mar 23;12(2):94–104. doi: 10.1080/21501203.2020.1740409 (PMC8128174; doi:10.1080/21501203.2020.1740409)
Supplement: Supplemental Material [file TMYC_A_1740409_SM9540.docx]

**Fig. S1.** The concentration of plasma glucose in rats before the treatment of *G. lucidum* supplement high-cholesterol diets in oral glucose tolerance test (OGTT). The rats were injected with nicotinamide and streptozotocin for the induction of type 2 diabetes as described in **Materials and methods**. After 1 week, the blood samples were collected at 120 min after glucose administration. The concentration of plasma glucose was measured by enzymatic kit following the supplier’s instruction. Results are expressed as mean ± SEM for each group of rats (n=8). ^#^p < 0.05 compared with the normal control group (NC)).

Table S1. Contents of mycelium and extracellular polysaccharides in the submerged culture of *Ganoderma lucidum* at 30^o^C for 7 days.

| **Item** | **Mycelium (g/L)** | **Extracellular Polysaccharides (g/L)** |
| --- | --- | --- |
| *Ganoderma lucidum* | 6.56 ± 0.74 | 0.50 ± 0.08 |

Results are expressed as mean ± SD for *Ganoderma lucidum* (n=3).
